# Performance Optimization of Marine Science and Numerical Modeling on HPC Cluster

**DOI:** 10.1371/journal.pone.0169130

**Published:** 2017-01-03

**Authors:** Dongdong Yang, Hailong Yang, Luming Wang, Yucong Zhou, Zhiyuan Zhang, Rui Wang, Yi Liu

**Affiliations:** 1 School of Computer Science and Engineering, Beihang University, Beijing, China; 2 Department of Computer Science and Technology, Tsinghua University, Beijing, China; 3 State Key Lab of Mathematical Engineering and Advanced Computing, Wuxi, China; Tianjin University, CHINA

## Abstract

Marine science and numerical modeling (MASNUM) is widely used in forecasting ocean wave movement, through simulating the variation tendency of the ocean wave. Although efforts have been devoted to improve the performance of MASNUM from various aspects by existing work, there is still large space unexplored for further performance improvement. In this paper, we aim at improving the performance of propagation solver and data access during the simulation, in addition to the efficiency of output I/O and load balance. Our optimizations include several effective techniques such as the algorithm redesign, load distribution optimization, parallel I/O and data access optimization. The experimental results demonstrate that our approach achieves higher performance compared to the state-of-the-art work, about 3.5x speedup without degrading the prediction accuracy. In addition, the parameter sensitivity analysis shows our optimizations are effective under various topography resolutions and output frequencies.

## Introduction

The ability to understand the climate patterns and predict the climate changes is critical to organize daily activities in human society. To study the complicated earth climate, various models are proposed to represent different layers of the earth such as atmosphere model [1–3], ocean model [4, 5], land model [6, 7] and sea ice model [8, 9]. These models are coupled [10, 11] together to simulate the climate phenomenon systematically. Since ocean takes three quarters of the earth surface, it has been an essential task for climate researchers to study the ocean activities in numerical models. MASNUM [12] is developed to simulate the progress of wave growth and propagation, which is the most common phenomenon in the ocean. Due to its advanced accuracy in both general and high sea state [13, 14], MASNUM is widely adopted in forecasting products.

MASNUM utilizes a global wave numerical model in spherical coordinates solving several important physical equations to simulate the wave activities. These equations [15] include breaking dissipation source equations, wave energy spectrum balance equations and complicated characteristic equations. Solving these equations in a reasonable time usually requires large amount of computing resources from a HPC cluster. Moreover, as the modern forecasting products pushing in the direction of higher resolution, the computation need of MASNUM grows significantly, which in turn generates increasing demand for HPC cluster.

In the meanwhile, the rapid development of high performance computers is pushing the edge of the climate research. The massive computing resources and scalable design of HPC cluster facilitate the demand for wave simulation with higher resolution. However, there is a large obstacle for climate researchers to fully utilize the capability of HPC cluster due to its underlying complexity. The massively parallel nature of HPC cluster requires a fundamental re-design of the wave model from various aspects including algorithm, load balance, process communication, I/O and etc.

Tremendous efforts have been devoted to the higher performance of ocean wave simulation. In [16], a parallel version of MASNUM using MPI was developed, improving the performance of the serial version. Later Zhang et al. [17] optimized the aggregation, data distribution and local blocking method to achieve higher performance. In [18] Zhao et al. improved parallel efficiency based on an irregular quasi-rectangular domain decomposition scheme in MASNUM. Later, Zhang et al. [19] proposed a way to optimize the load balance by keeping the amount of computation in each process close to the mean. In addition, they designed a new I/O strategy to improve the I/O performance as well as MPI message packing method to reduce the communication overhead. Inspired by [19], our work takes a deep dive to further optimize the performance of MASNUM from multiple aspects such as the algorithms, communication pattern, load balance and data accessing.

Although the performance of MASNUM has been improved by previous work, we find there is still large space unexplored for further optimization. For example, redundant calculation and constrained I/O are all potential directions for performance optimization. To improve the parallel efficiency as well as reduce the complexity of computation, we propose several performance optimizations of MASNUM running on a cluster. Specifically, this paper makes the following contributions:

- We develop two methods to accelerate the bottleneck functions through algorithm redesign and redundant calculation elimination.
- We design a better strategy for process communication and load balance with improved performance.
- We parallelize output I/O that significantly reduces the I/O delay at high output frequency.
- We enhance the data locality and alignment for improved cache hit ratio both temporally and spatially.
- We demonstrate a 3.5x speedup of MASNUM in high topography resolution after applying the proposed optimizations.

The remainder of this paper is organized as follows. Section “Bottleneck Analysis” analyzes the bottlenecks of current MASNUM implementation. Section “Design Decisions for Optimizations” discusses the design decisions for optimizing MASNUM from several aspects. Section “Evaluation” presents the experimental results with comparison to the previous work. Finally, related work and conclusions are presented in Section “Related Work” and “Conclusion”, respectively.

## Bottleneck Analysis

**Fig 1:**
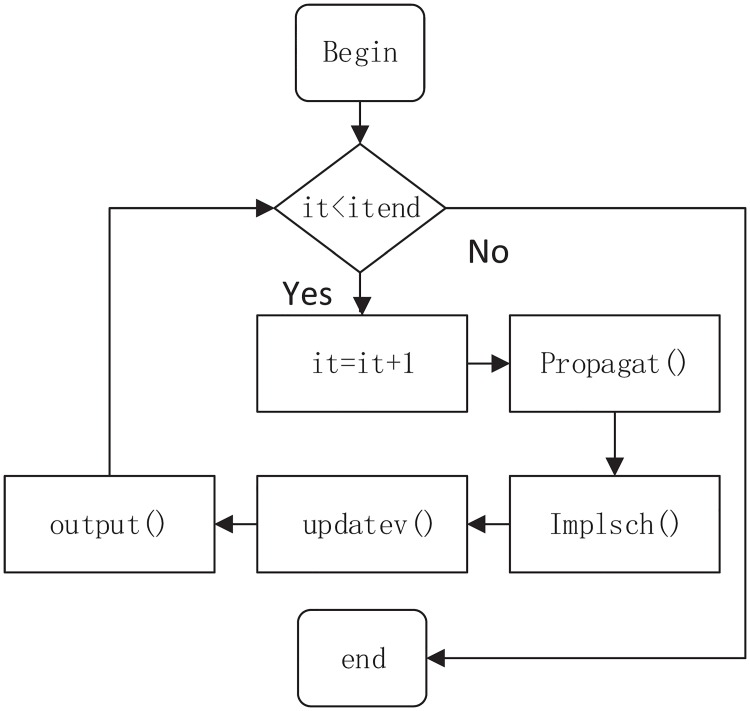
The simplified program structure and execution flow of MASNUM.

The whole MUSNUM program contains five critical functions such as *propagat*, *implsch*, *mean1*, *updatev* and *output*, as shown in Fig 1. The purpose of each function is listed as follows:

- *propagat* is to solve the spread of wave equations.
- *implsch* is to solve the local changes caused by the source functions.
- *mean1* is to solve the characteristic of the wave equations.
- *updatev* is for the communication among adjacent blocks.
- *output* is to write the simulation results into files.

**Fig 2:**
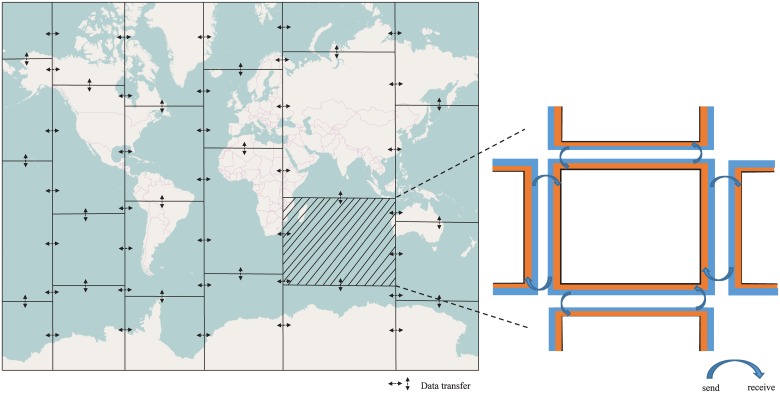
Process communication pattern. Process communication among adjacent matrixes to update data during MASNUM simulation.

During the simulation, MASNUM divides the input matrix into small blocks, and then distributes them into different processes according to the theory of Jacobi matrix’s iteration (e.g., 4 × 6 blocks with 24 processes). The process communication pattern of MASNUM is illustrated in Fig 2. There are halo regions (rectangles shown at the right side of Fig 2) at the block boundary of each process, which buffer the data to exchange with its neighbor. Before each iteration starts, the process in charge of a particular block sends the data in the halo region to its neighbor, as well as fetches the data into the halo region from its neighbor. After the data exchanges, each process computes the data in the assigned block and update the halo region independently. The computation of Jacobi iteration in MASNUM is quite effective, especially when the simulation is done in large scale.

**Fig 3:**
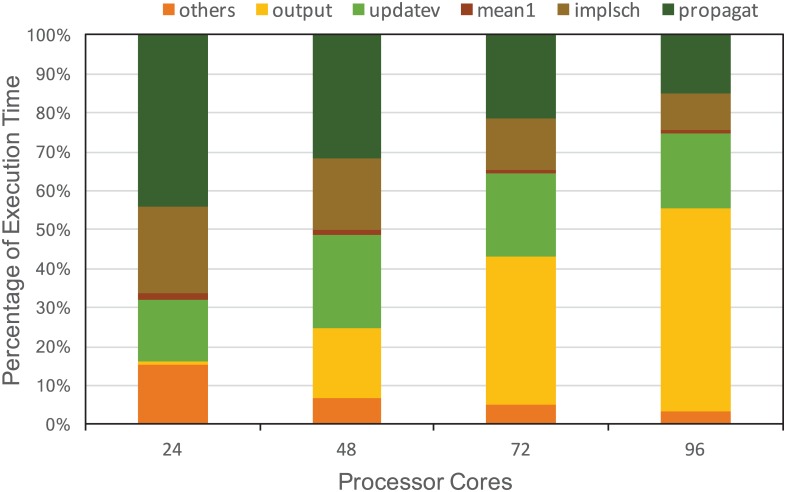
Performance profiling. The time fraction of critical functions at each time step.

To identify the bottleneck functions of MASNUM, we analyze the fraction of the execution time that each critical function takes at one time step. The time fraction is shown in Fig 3, profiled by Intel VTune Amplifier. The experiment simulates the western Pacific (100-150E, 10S-60N) ocean surface wave. The simulation time is from 2009-01-01 to 2009-01-04, with the time step of 5 minutes. The resolution is 1/2 degree and the output frequency is once per 24 hours. We allow each processor core in our experiment to run only one process. As seen in Fig 3, the bottleneck functions change as more processor core used. For instance, when the cores number is 24, we observe that propagat (54.10%), implsch (22.54%) are the two major bottleneck functions. As the number of cores increases, the bottleneck of MASNUM shifts to I/O function *output*. Generally, when the number of cores increases to 96, the percentage of the total execution time due to I/O is nearly 50%. At the same time, the percentage of total execution time in communication stays around 20% across all core scales. Thus we treat the computation (*propagat*), I/O (*output*) and communication (*updatev*) as the bottleneck functions of current MASNUM implementation and provide detailed analysis accordingly to reveal the opportunities for optimization.

### Propagation Solver

The propagation subroutine calculates the location *X*_0_, wave-number *K*_0_ of the wave packet at time *t* − Δ*t* which propagates to location *X* at time *t* and obtains wave-number *K* by solving the complicated characteristic equations. Therefore we get the responding spectrum *E*(*K*_0_, *X*_0_, *t* − Δ*t*) at time *t* from interpolation in phase and physical space. The goal is to calculate the wave energy-current spreading, and then to gather the effect of refraction caused by topography and current. Eventually, the wave energy at the physical space point and the wave space point is determined. The most time-consuming portion of the propagation subroutine is to solve the propagation equation, and the solution is shown in Eqs 1 and 2, where *λ* is the longitude, *φ* is the latitude, *t* is the time step, *R* is the radius of the earth, *C*_*gλ*_ denotes the group velocity in the direction of longitude *φ*, *C*_*gφ*_ represents the group velocity in the direction of latitude *λ* and *U*_*φ*_ denotes the velocity in the direction of longitude *φ*. For detailed proof, readers can refer to [15].

$$\begin{array}{c} \frac{d\lambda }{dt}=\frac{C_{g\lambda }+U_{\lambda }}{Rcos\varphi } \end{array}$$ (1)

$$\begin{array}{c} \frac{d\varphi }{dt}=\frac{C_{g\varphi }+U_{\varphi }}{R} \end{array}$$ (2)

### Load Distribution and Communication

Considering the land-sea distribution, the original MASNUM implementation takes the following procedures to assign load across processes. First, dividing the whole input matrix into *x*_*proc*_ columns and then into *y*_*proc*_ rows, where *x*_*proc*_ is the number of processes along the longitude and *y*_*proc*_ is the number of processes along the latitude. The Algorithm 1 first distributes all of the grids into *x*_*proc*_ rows, and the total number of grids is no less than the average load. Then the algorithm distributes all of the grid in each row into *y*_*proc*_ columns. This distribution strategy leads imbalanced load of processes along the rows and columns. For instance, the load of last column and last row is far less than the average. The imbalanced load distribution is also illustrated in Fig 2, where the small cell in the grid represents less load for computation and the large cell means the opposite.

To improve the load imbalance of MASNUM, Zhang et al. [19] modified the load distribution strategy in step (4) and (10) of Algorithm 1, which makes *N*_*i*_ close to *N*_*avg*_. Their strategy for load distribution proceeds as follows. If *N*_*i*_ is less than *N*_*avg*_, and *N*_*avg*_ equals $\frac{N_{already-i}}{x_{already-i}-1}$, *N*_*already*−*i*_ represents the total number of distributed grid so far, where *x*_*already*−*i*_ is the index of matrix waiting for being distributed. The load distributed along the latitude is the same as along the longitude. However, we find that keeping each process with approximately equal load is not always the best strategy for MASNUM. The reason is that the root process always does more work in reading and writing the data in the format of NetCDF file [20]. If the simulation output frequency is high, it is better to keep the computation of the root process lower than the average. Furthermore, we find that previous load distribution strategy neglects the heterogeneous communication patterns in different directions, wasting the potential opportunity for further optimization. For instance, along longitude direction the communication is non-blocking while along latitude direction it is blocking.

**Algorithm 1** The load distribution strategy of original MASNUM

1: //*N*_*total*_ is the total grid number of the input matrix. *x*_*proc*_ is the number of processes along the longitude and *y*_*proc*_ is along the latitude

2: **for** *i* = 0 to *x*_*proc*_ − 2 **do**

3:  *N*_*avg*_ = *N*_*total*_ / *x*_*proc*_

4:  Distributes *N*_*i*_ to the processes with column index *i*, where *N*_*i*_ is no less than *N*_*avg*_

5: **end for**

6: Distributes the remaining to the number of *x*_*proc*_ − 1

7: **for** *i* = 0 to *x*_*proc*_ − 1 **do**

8:  **for** *j* = 0 to *y*_*proc*_ − 2 **do**

9:   *N*_*avg*_ = *N*_*i*_ / *y*_*proc*_

10:   Distributes *N*_*j*_ to the processes with row index *j*, *N*_*j*_ is no less than *N*_*avg*_

11:  **end for**

12:  Distributes the remaining to the number of *y*_*proc*_ − 1

13: **end for**

### I/O Strategy

**Fig 4:**
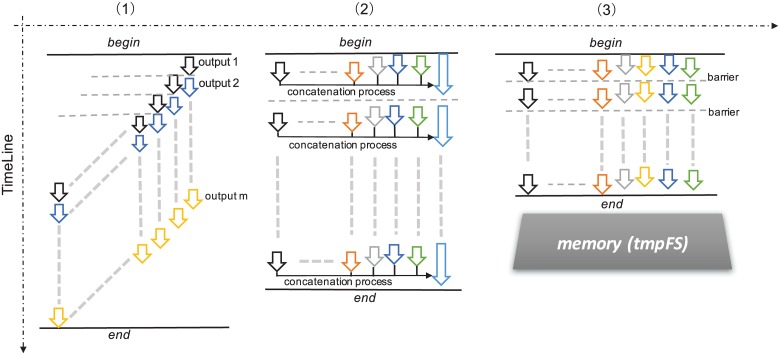
The I/O strategies when writing the output data. (1) sequential write: one process writes the output at a time, (2) parallel write: multiple processes write the output concurrently, (3) parallel write + memory filesystem: store the output into memory filesystem temporally to circumvent disk access.

After analyzing the I/O of MASNUM, we find the simulation output is written by the processes sequentially, as shown in Fig 4(1). Only when the process ahead of the current one finishes its output progress, could the current one begin to work. Therefore, as the number of the processes scales, the program requires longer time to generate the output. Zhang et al. [19] proposed to buffer the output from other processes into memory in parallel and then allow the root process to write the buffer into disk, as shown in Fig 4(2). Although this technique alleviates the sequential bottleneck, the I/O performance is still limited by a single write process. In addition, buffering data into memory is not feasible when the input matrix is too large. Moreover, when the root process is writing data into disk, other processes stay idle. In contrast, we advocate to eliminate the intrinsic serial I/O and enable each process to write the output concurrently, which boosts the I/O performance fundamentally.

### Cache Locality

During the performance profiling of MASNUM, we notice that the average cache miss rate is high, with 32.72% of all cache references and 31.63% of last level cache (LLC), although the L1 data load miss is quite low of 0.24%. After analyzing the program, we find that some data structures such as *ee* (wave spectrum) and *e* (wave spectrum through propagation) arrays are visited by line rather than by column. In addition, a large number of data is generated during the simulation, however not used until a long reuse distance, both of which results in the low cache hit ratio.

## Design Decisions for Optimizations

In this section, we focus on optimization techniques applied to MASNUM. Section “‘Algorithm Optimization” presents the algorithm optimization to reduce the amount of computation. Section “‘Load Distribution Optimization” details the load distribution optimization. Parallel I/O and data access improvement is presented in section “‘Parallel I/O” and “‘Data Access Optimization” respectively.

### Algorithm Optimization

#### Optimizing Searching Method via Derivation Feature

The searching process within the propagation function is to find the index of longitude in order to solve the Eqs 1 and 2.

**Fig 5:**
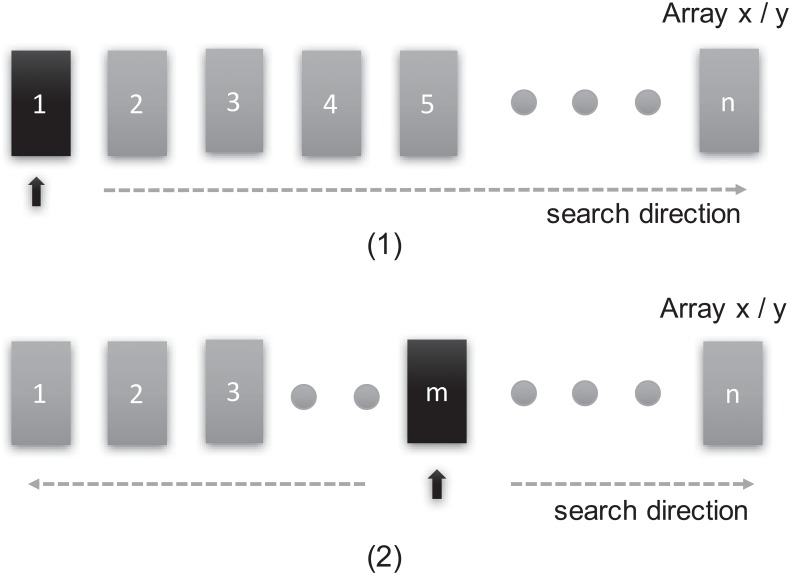
Searching method. The models of original traversal searching method and optimized searching method via derivation feature.

Fig 5(1) illustrates how the original search algorithm finds the right position in each iteration in the direction of longitude. The begin position of the original algorithm is zero as shown in Fig 5(1) with the orange square. Assuming the length of the input grid is *L*, the time complexity of *propagat* function is *O*(*N* ⋅ *L*), where *N* denotes the number of the grid. Fig 5(2) shows our optimization, which finds the right position by leveraging the result of the last iteration. For instance, the begin position, which is the orange square in Fig 5(2), is the position of *m* after the last iteration.

In the search algorithm, *x* is the array of longitude (from 180°*W* to 180°*E*) in ascending order. Based on the integral Eqs 1 and 2, the changes of *λ* and *φ* are usually small when the two points are adjacent, unless the topography and the wind change radically. Thus we leverage this specific feature to approximate adjacent points with the same value, reducing the complexity of computation. With this optimization, the algorithm complexity tends to *O*(1), although the worst case is *O*(*L*), which is the same as the original algorithm. Therefore, the time complexity of *propagat* function tends to *O*(*N*). The same optimization is applied to array *y*, which stores the input data along the latitude (from 90°*S* to 90°*N*).

#### Store the Intermediate Results in Inner Loop

As Algorithm 2 shows, the variables of *ia* and *ic* share the same value between loop *j* and *k*. Therefore we store the value of these two variables in the inner loop whenever they are calculated, which is reused for further computation. Storing the intermediate results is able to reduce the amount of computation effectively and thus speedup the performance of the algorithm. Moreover, the trigonometric function within loop *k* is time-consuming and do not need to be calculated repeatedly. Therefore, we store the result of the trigonometric function whenever it is calculated and return the result directly when it is accessed afterward.

**Algorithm 2** Store the Calculated Results

1: //*R* represents the size of each block, Ω is grid indexes in wave-number space, *j* is wave-number spectrum and *k* is wave-number of Ω

2: **for** all point (*ia*, *ic*) ∈ *R* **do**

3:  **loop** *j*

4:   **if** *J*_*flag*_ == 0 **then**                ▷ Loop j

5:    Data dependent of (*ia*, *ic*)

6:    Store those data into temporary matrix *T*_1_

7:    *J*_*flag*_ = 1

8:   **else**

9:    Read the temporary matrix *T*_1_

10:   **end if**

11:   **look** *k*

12:    **if** *K*_*flag*_ == 0 **then**               ▷ Loop k

13:     Data dependent of (*ia*, *ic*) and loop *j*

14:     Store data into temporary matrix *T*_2_

15:     *K*_*flag*_ = 1

16:    **else**

17:     Read the temporary matrix *T*_2_

18:    **end if**

19:   **end loop**

20:   *K*_*flag*_ = 0

21:  **end loop**

22:  *J*_*flag*_ = 0

23: **end for**

### Load Distribution Optimization

Let *x*_*proc*_ and *y*_*proc*_ denote the number of processes along the longitude and latitude direction respectively. The relationship between *x*_*proc*_ and *y*_*proc*_ is depicted in Eq 3, where *n* is the total number of processes. Since for each pair of layout, there are always two options for *x*_*proc*_ and *y*_*proc*_. For instance, if the total number of processes is 24, we could set the layout to be 4 × 6 or the other way around.

$$\begin{array}{c} nx=\sqrt{n\cdot \frac{x}{y}} \end{array}$$ (3)

**Fig 6:**
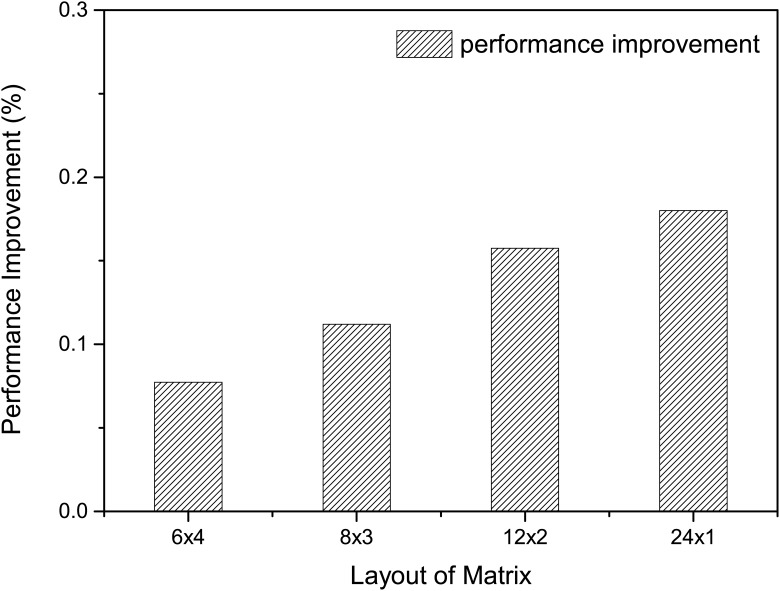
Impact of layout on performance. The performance variance under different layouts on 24 processor cores. The result is normalized to its layout counterpart.

To better understand how different process layouts affect the performance, we experiment with several different layouts (4 × 6, 6 × 4, 3 × 8, 8 × 3, 2 × 12, 12 × 2, 1 × 24 and 24 × 1) on 24 processor cores regarding the same input and simulation settings. The performance variance is shown in Fig 6. The result is normalized to the layout in its counterpart. For instance, compared to the layout of 4 × 6, the opposite layout improves the performance by almost 8%. The same tendency is observed across all possible layouts in Fig 6, that assigning more processes along the *x* direction than *y* direction always leads to better performance. Especially, the performance benefit becomes larger as the numerical difference between *x* and *y* increases.

**Fig 7:**
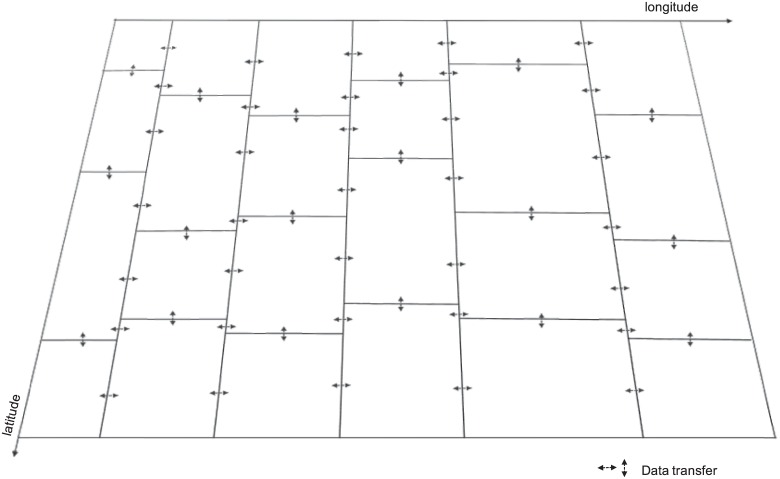
An example layout of processes. 6 × 4 layout of processes with 24 processor cores.

Based on the above observation, we propose to use more processes along the longitude than latitude direction in order to improve the performance. In addition, the MASNUM program is implemented using *fortran* language, with which the arrays are accessed by column first instead of row. Therefore, the proposed optimization also increases the cache hit ratio since more processes are accessing data in the longitude (column) direction. Moreover, for each grid block, it is easier to communicate with grid blocks in the longitude direction than the ones in the latitude direction. As shown in Fig 7, this is because the data transferring is non-blocking in the longitude direction, whereas blocking in the latitude direction. Thus assigning more processes in the latitude direction reduces the waiting time during the data transferring.

Considering that root process performs more work than other processes, specifically generating the NetCDF files and initiating the attributes. We improve the load distribution algorithm in previous work [19] through incorporating a control parameter *δ* as shown in Algorithm 3. In the meanwhile, based on the observations in Fig 6, we assign more processes along the longitude (*x*_*proc*_) than latitude (*y*_*proc*_) direction, optimizing the communication between adjacent grid blocks.

**Algorithm 3** Load Distribution Algorithm

1: // *x*_*proc*_ is no less than *y*_*proc*_

2: *N*_*avg*_ = *N*_*total*_ / (*x*_*proc*_ * *y*_*proc*_)

3: *N*_0_ = δ*N_*avg*_

4: *N*_*all_avg*_ = (*N*_*total*_ − *N*_0_)/(*x*_*proc*_ * *y*_*proc*_ − 1)

5: **for** *i* = 0 to *x*_*proc*_ − 2 **do**

6:  *N*_*avg*_ is the average of distributed dots

7:  Distributes *N*_*i*_ to the column of processes with column index *i*, *N*_*i*_ is not less than *N*_*avg*_ if *N*_*avg*_ < *N*_*all*_*avg*_, or not more than *N*_*avg*_ if *N*_*avg*_ > *N*_*all*_*avg*_

8: **end for**

9: Distributes the remaining to the number of *x*_*proc*_ − 1

10: **for** *i* = 0 to *x*_*proc*_ − 1 **do**

11:  **for** *j* = 0 to *y*_*proc*_ − 2 **do**

12:   **if** *i* == 0 & *j* == 0 **then**

13:    continue:

14:   **end if**

15:   *N*_*avg*_ is the average of distributed dots in *N*_*i*_

16:   Distributes *N*_*j*_ to the column of processes with column index *j*, *N*_*j*_ is not less than *N*_*avg*_ if *N*_*avg*_ < *N*_*all*_*avg*_, or not more than *N*_*avg*_ if *N*_*avg*_ > *N*_*all*_*avg*_

17:  **end for**

18:  Distributes the remaining to the number of *y*_*proc*_ − 1

19: **end for**

### Parallel I/O

As illustrated in section “‘Bottleneck Analysis”, sequential I/O becomes a bottleneck as the number of processes increases. The more processes there are, the longer time it takes for the processes to write the output file sequentially. Although Zhang et al. [19] proposed to buffer the output and use the root process to write into the disk on behavior of all processes, the performance is still limited by the I/O especially when the volume of output becomes large. Instead, we replace the sequential I/O by leveraging the library of parallel NetCDF [21], which allows multiple processes to perform I/O simultaneously. In addition, to further speedup the performance during the output stage, we use an in-memory file system called tmpFS [22] that eliminates the access to hard disk as shown in Fig 4(3).

### Data Access Optimization

#### Data locality

Due to the cache effect, accessing data in near locations improves the cache hit ratio. Therefore, we optimize the MASNUM program so that the variables calculated in each time step would be accessed in the near future as much as possible. Since the array in *Fortran* is organized in column major order, it leads to higher cache hit ratio if the array is visited by column. We changed the data access pattern in several functions such as the *propagat*, *implsch*, *mean1* and *readwi_mpi*, so that the data locality is improved during the computaion.

#### Data alignment

Data alignment is important especially when leveraging the compiler techniques such as auto-vectorization (with *-xhost* option). Unaligned accesses lead to ineffective load and store operations in memory. Thus, we align the data accesses for better performance by using pragmas and directives. In MASNUM, most data is stored in the form of multi-dimension arrays. We set the multi-dimension arrays with 64-byte boundary to perform memory accesses more efficiently on Intel Xeon processor.

## Evaluation

### Experimental Setup

The node configuration of our experiment cluster is shown in Table 1. The cluster is composed of 8 homogenous nodes. We evaluate the performance of MASNUM at different scales when applying the optimizations we proposed. The performance is measured as execution time. According to our knowledge, [19] proposed the most recent performance optimization on MASNUM. Therefore, we use [19] as our baseline throughout the evaluation. For the ease of comparison, we provide the percent of improvement over [19]. The simulation is setup to predict the ocean surface wave of the western Pacific. The simulation time is four days long with the time step of five minutes. The topography resolution is 0.2 degree. The rest of the simulation parameters keep the default. All the following experiments use the same simulation setup unless specifically mentioned.

**Table 1:** Node Configuration of the Experiment Cluster.

Configuration	Setting
CPU	2 × Intel Xeon E5-2680v3@2.5 GHz(12 cores)
Memory	128GB DDR4
Hard Disk	2 × 300GB SSD
MPI	Intel MPI Version 5.0.3
Network	Mellanox InfiniBand
Parallel NetCDF	Version 1.7.0
Operation System	CentOS 7.2 x86_64
MASNUM	Version 2.2

### Overall Performance Improvement

**Fig 8:**
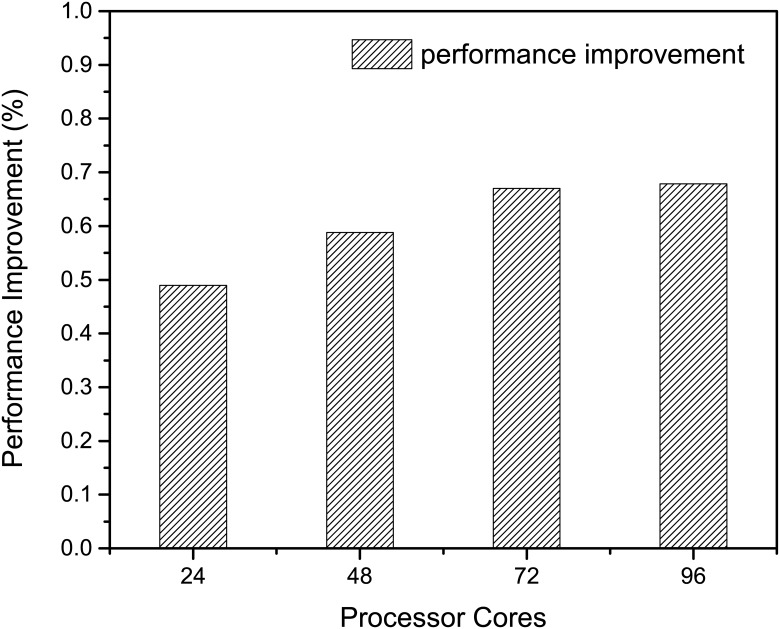
Overall performance improvement. Performance improvement of MASNUM after applying all the optimizations proposed.

**Fig 9:**
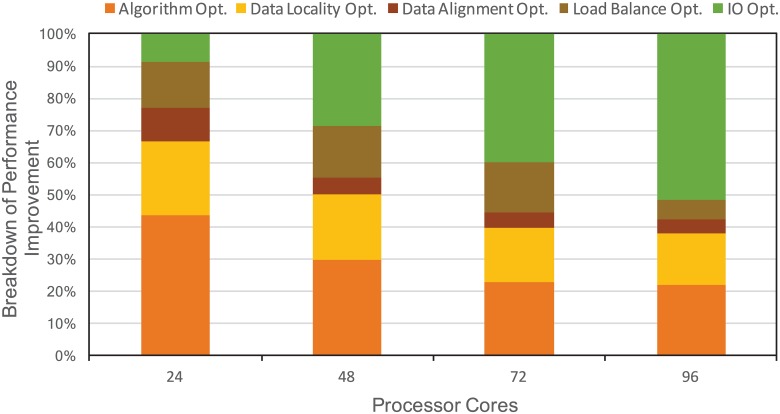
Breakdown of performance improvement. The percentage of performance improvement of MASNUM that each of the proposed optimizations contributes to.

First, we evaluate the overall performance improvement of MASNUM after applying all the optimizations we proposed. As shown in Fig 8, the overall performance improvement is quite significant in all scales, ranging from 49% to 68%. It is also noticed the performance improvement scales linearly as the number of cores increases from 24 to 72. Although when the number of cores goes beyond 72, the performance improvement stays almost constant around 67.6%. The experiments demonstrate the effectiveness of our proposed optimization in improving the performance of MASNUM. In addition, we show in Fig 9 the breakdown of performance improvement that each proposed optimization contributes to. The algorithm optimization contributes most to the overall performance improvement when running MASNUM at small scale, by 43.9% at 24 cores. However, I/O optimization becomes dominate when the number of cores scales, by 51.7% at 96 cores. We also notice that data access optimization including data locality and data alignment optimizations takes an important portion of the overall performance improvement, ranging from 33.3% to 20.7%. The detailed evaluation of each optimization is provided in the following sections.

### Algorithm Optimization

**Fig 10:**
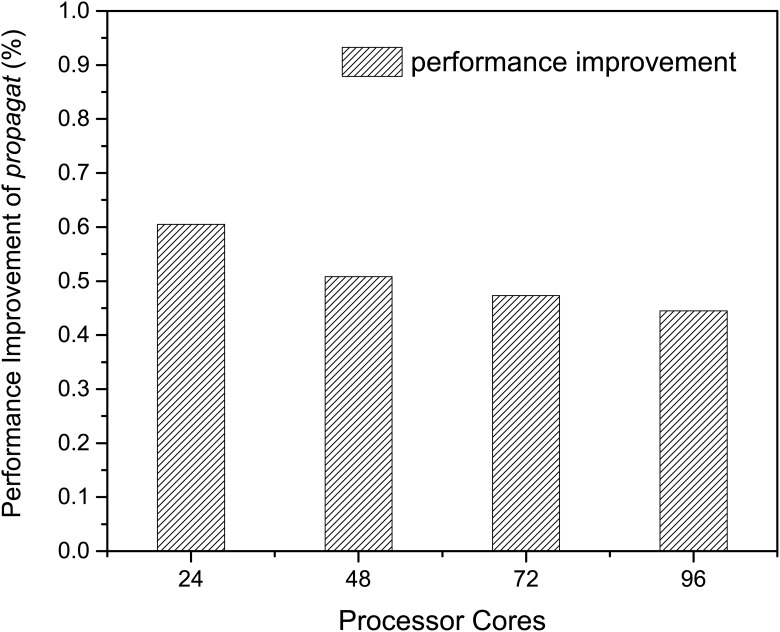
Performance improvement from algorithm optimization. The performance improvement of function *propagat* using algorithm optimization.

In Fig 10, the performance of propagation function is improved by more than 44% at all scales. The best performance improvement is achieved with 24 cores by 60%. In fact, our optimization for the searching method is more effective than binary search. For binary search, the time complexity is *O*(*logL*), where *L* is the length of the array of longitude or latitude. The drawback of binary search is that it fails to leverage the ordered elements in the arrays, which our optimization takes advantage of. Thus our optimization on searching algorithm always performs better than binary search. The slowed down performance improvement as the number of cores increases is because as more processes are launched, the less amount of work is assigned to the propagation function in each process, which diminishes the improvement of our algorithm optimization. In general, our algorithm optimization is quite effective to improve the performance of propagation function.

### Load Distribution and Data Access Optimization

**Fig 11:**
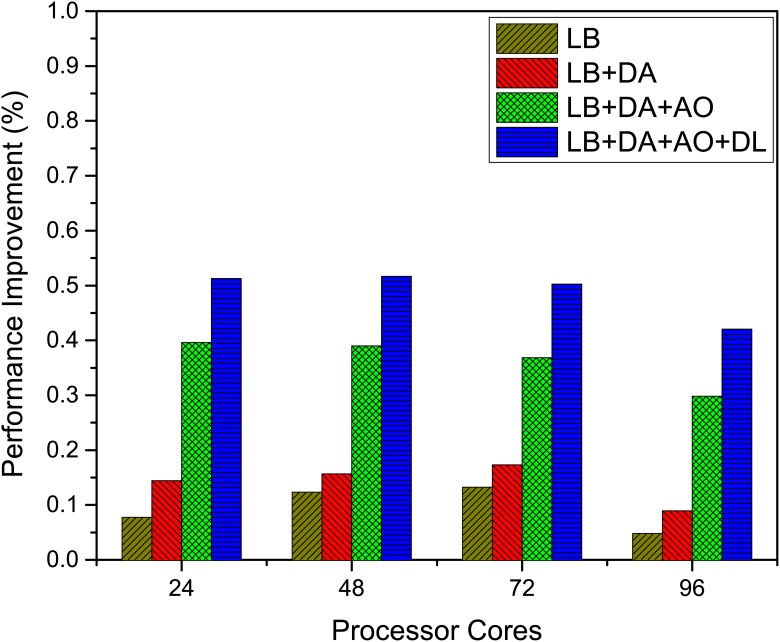
Performance improvement from load distribution and data access optimization. The performance improvement of MASNUM using different optimization methods, where LB stands for load balance, DA stands for data alignment, AO stands for algorithm optimization and DL stands for data locality.

As shown in Fig 11, after applying the load balance optimization (LB), we achieve 8% to 13% performance improvement as the number of cores scales from 24 to 72. In addition to LB optimization, data alignment optimization (DA) gives another 5% performance improvement at all scales. The best performance improvement is achieved when combining the algorithm optimization (AO) from previous section, which increases the performance by 25% at 24 cores in addition to the former two optimizations. Furthermore, data locality optimization (DL) boosts the performance by additional 10% at all scales over the combination of LB, DA and AO. It is noticed that after the number of cores increases beyond 72, the percentage of performance improvement decreases apparently. The reason can be explained as the number of cores increases, the amount of work assigned to each core becomes less, which undermines the effectiveness of all optimizations. With all optimizations, the percentage of performance improvement ranges from 43% to 52%.

### I/O Optimization

**Fig 12:**
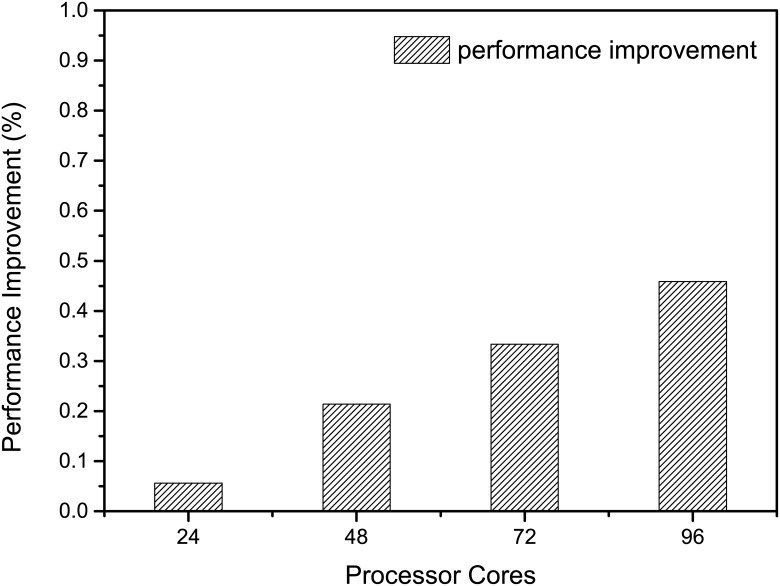
Performance improvement from I/O optimization. The performance improvement of MASNUM using I/O optimization.

The performance improvement through I/O optimization is shown in Fig 12. As the number of cores increases from 24 to 96, the performance improvement scales linearly from 5.6% to 45.9%. The linear scaling property of I/O optimization is due to the private MPI communication field we create each time to receive the output data. In addition, to avoid the overhead of initialization by multiple processes, only one process takes charge of creating the output file and setting up initial attributes. Thus with more processes created, the performance improves accordingly.

### Parameter Sensitivity

To investigate the sensitivity of our optimizations under different parameter settings, we design experiments to evaluate the performance improvement by changing one parameter at a time while keeping the rest constant. Identified by our empirical study, two parameters including topography resolution and output frequency show strong impact on the performance of MASNUM simulation. During the sensitivity experiment of topography resolution, we change the resolution to be 0.125, 0.2, 0.4 and 0.5 degrees respectively. The smaller degree means higher resolution. Similarly, to evaluate the sensitivity of the output frequency, it is set to one hour, two hours, six hours and 24 hours respectively. The longer hours means lower output frequency.

#### Topography Resolution

**Fig 13:**
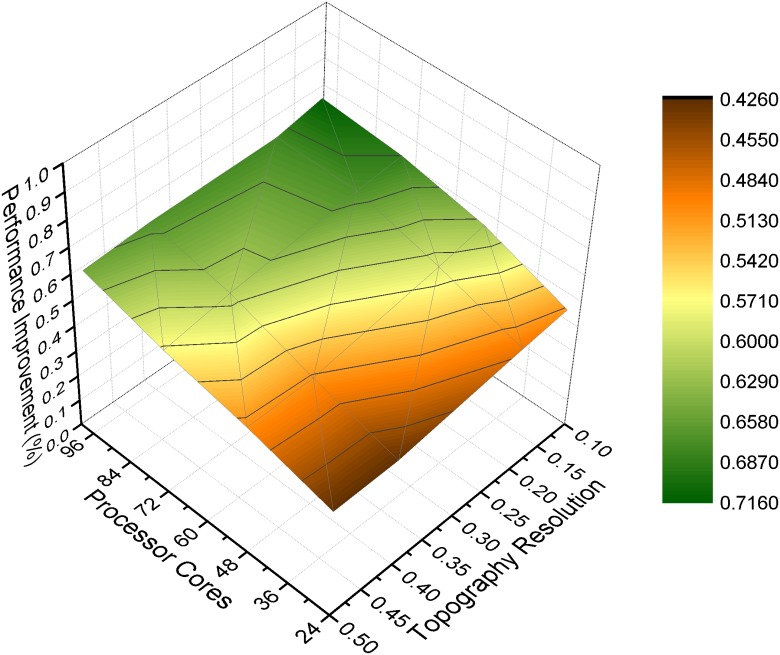
Parameter sensitivity of topography resolution. The performance improvement under different topography resolutions.

As Fig 13 shows, the performance improvement increases when the input resolution becomes higher. This tendency scales as more cores are used in the simulation. The most performance improvement is 71.6% (3.5x speedup), which is achieved at 96 cores with the input resolution of 0.125 degree. The reason for the higher performance at higher resolution can be attributed to I/O optimization we proposed. At high resolution, each process needs to generate more data for the output, deteriorating the performance of previous work with serial I/O. In our I/O optimization, the serial I/O is eliminated by using the library of *PnetCDF*, which allows multiple processes to write the output file simultaneously. The experiments demonstrate our optimizations are capable of improving the performance with increasing topography resolution.

#### Output Frequency

**Fig 14:**
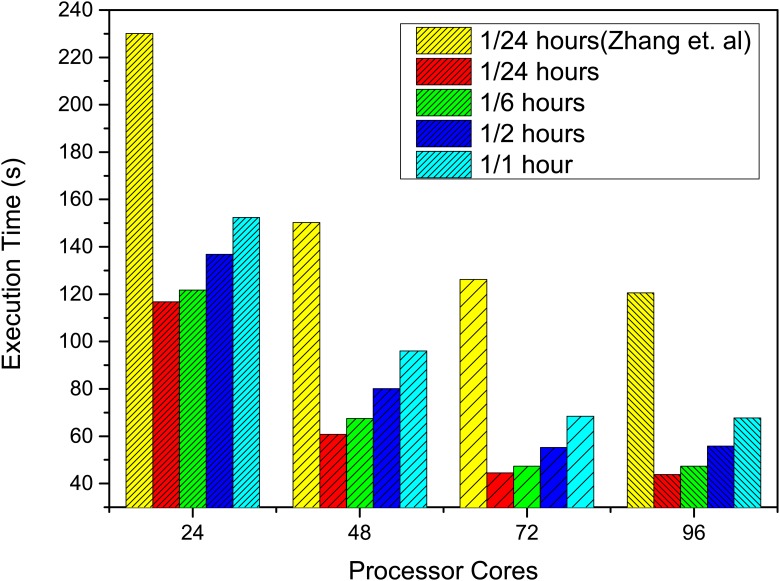
Parameter sensitivity of output frequency. The performance improvement under different output frequency.

The sensitivity analysis on output frequency is illustrated in Fig 14. Due to the extremely long execution time with the original implementation, we only include the performance comparison at the output frequency of one per 24 hours. In the comparison, the performance is improved by 47.8% under all scales. As the output frequency increases from one per 24 hours to one per hour, the execution time increases by 30% universally. The prolonged execution time can be attributed to the larger amount of I/O under higher output frequency. With the core number increasing from 24 to 96, the performance is improved by 63.6% at all output frequencies. This demonstrates that our optimizations achieve significant performance improvement under various output frequencies.

### Prediction Accuracy

Due to the round-off difference from the calculation of wave energy spreading and the chaotic nature of the wave dynamics, it is infeasible to provide bit-for-bit (BFB) identical results in wave simulations. To verify the accuracy of the MASNUM simulation after applying our optimizations, we calculate the root-mean-square deviation (RMSD) [23] of the results given before and after the optimizations. The equation of RMSD is defined in Eq 4, where *X*_0_ and *X* represent the results before and after optimizations, *n* represents the number of sampling. At a given point *i*, there are series of observations for each variable and *k* defines the number of variables under observation. Inspired by [24], we validate six representative variables such as zonal wind velocity (*windx*), meridional wind velocity (*windy*), significant wave height (*hs*), mean wave direction (*th*), spectrum peak wave period (*tp*) and zero-crossing wave period (*tz*). Thus *k* ranges from 1 to 6.

$$\begin{array}{c} RMSD\left(X,X_{0},k\right)=\sqrt{\frac{1}{n}\sum_{i=1}^{n}{\left(X_{ik}-X_{0ik}\right)}^{2}} \end{array}$$ (4)

**Fig 15:**
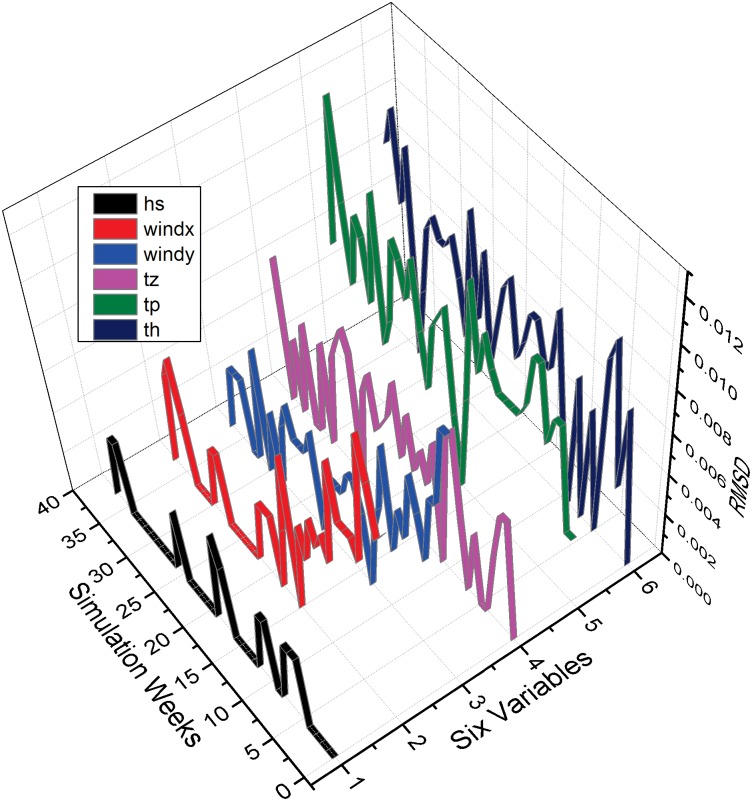
Validation of accuracy. The accuracy of MASNUM after applying the proposed optimizations. The validation is against six important variables in MASNUM.

In Fig 15, we simulate 38 weeks to verify the accuracy of the above six variables across the time-line. The rest of the simulation parameters are the same as those in section “‘Experimental Setup”. As shown, the RMSD across all six variables is lower than 0.012 during the whole simulation, which indicates the proposed optimizations do not introduce additional inaccuracy to the simulation. For instance, the variable *hs* representing the significant wave height exhibits the smallest RDSM of less than 0.005.

## Related Work

We briefly review the related work from two categories: general efforts to improve the ocean modeling performance and specific ones on the wave modeling. In the first category, for reducing the communication overhead, OpenMP is demonstrated to be effective in improving modeling performance at large core count [25]. Land elimination is another approach to reducing the communication overhead and in [26, 27], space-filling curves are used to improve load balance as well as to reduce the amount of communication. Additionally, in [28], the authors proposed to overlap the global communication with the matrix-vector product via a pipelined method that improved the performance of conjugate gradient. In [29], Hu et al. implemented a new solver that reduced the communication overhead as the core count increased, which lead to higher speedup at large scale. Although previous work is able to effectively improve the performance of ocean modeling, they cannot be directly applied to the optimization of wave modeling.

For the wave modeling, most of the existing work is developed from third generation numerical wave model [15, 30]. Specifically, the marine science and wave numerical model (MASNUM) was proposed and developed in spherical coordinates in theory [12, 31]. After that, several optimization work is proposed to wave modeling. In [16], the parallel version of MASNUM based on MPI was developed. Later, Zhang et al. [17] optimized the aggregation, data distribution and local blocking method to get higher performance. Since the matrix segmentation leads to poor load balance, Alamos National Laboratory introduced the notion of the smallest fundamental block [32, 33]. In their work, the size of the smallest block is determined first, and then each block is distributed into different regions. Each process is assigned an equal number of computation regions in order to achieve better load balance. Later in [18], Zhao et al. developed a highly efficient parallel numerical surface wave model based on an irregular quasi-rectangular domain decomposition.

Our work is closely related to Zhang et al. [19] which focused on load balance, communication and I/O optimization. In their load balance optimization, the amount of computation in each process was maintained close to the mean. They also proposed new I/O strategy to improve the I/O performance as well as a message packeting method to reduce the communication overhead. In comparison, our work optimizes MASNUM program from multiple aspects such as the algorithm, communication, parallel I/O and data locality, which provides us more opportunities to boost the performance further. We also notice that parallel framework MapReduce [34] has been applied to address the massive I/O problem in scientific computation such as multiple sequence alignment [35, 36].

## Conclusion

The demand of high-resolution MASNUM wave simulations has been driven the optimization work from different directions. This paper improves the performance of the propagation solver via reducing the redundant computations. In addition, we improve the efficiency of communication among processes during the computation. Furthermore, we eliminate the bottleneck of serial I/O during the output stage using parallel NetCDF. Finally, we enhance the data locality during the calculation for better cache hit ratio. Our proposed optimizations achieve 3.5x speedup compared to the state-of-the-art work, without degrading the prediction accuracy. The parameter sensitivity experiments demonstrate our optimizations are effective under various parameter settings.
